# Pannexin-1 Contributes to the Apoptosis of Spinal Neurocytes in Spinal Cord Injury

**DOI:** 10.3389/fphys.2021.656647

**Published:** 2021-04-27

**Authors:** Yu Huang, Jin Lin, Xuanwei Chen, Jianhua Lin

**Affiliations:** ^1^Department of Spine Surgery, The First Affiliated Hospital of Fujian Medical University, Fuzhou, China; ^2^Department of Basic Medical Science, Fujian Health College, Fuzhou, China

**Keywords:** pannexin-1 channels, spinal cord injury, apoptosis, Ca^2+^ influx, neurocytes

## Abstract

Currently, the role of Pannexin-1, a homomeric membrane hemichannel on the neuron cell membrane, in the development of spinal cord injury (SCI) is largely unknown. Herein, we assessed the contribution of Panx1 in the development of SCI. The SCI *in vitro* model was established using rat primary spinal neurocytes treated with hydrogen peroxide (H_2_O_2_). Effects of Panx1 overexpression or depletion in spinal neurocytes were analyzed by lentivirus-mediated transfection of Panx1 and interference sh-Panx1. Decreased cell viability was seen in SCI cells, which was further enhanced under Panx1 overexpression and mitigated by Panx1 deficiency. H_2_O_2_ induced an increase of intracellular Ca^2+^ signal and upregulated level of the proapoptotic protein Bax, and apoptosis pathway proteins including cleaved Caspase-3 and PARP1, which was enhanced by Panx1 overexpression or attenuated by Panx1 depletion. On the other hand, H_2_O_2_ treatment suppressed the level of antiapoptotic protein Bcl-2, which was further decreased by Panx1 overexpression or mitigated by Panx1 depletion. The results indicate that Panx1 was involved in the intracellular Ca^2+^ overload of SCI cells by accelerating extracellular Ca^2+^ influx, which promoted the apoptosis of spinal neurocytes through Ca^2+^ dependent pathways, thus aggravating the secondary injury of SCI.

## Introduction

Spinal cord injury (SCI) is one major cause of morbidity worldwide with impacts on quality of life and the healthcare system. The pathological outcomes of SCI mainly include damage to the white and gray matter of the spinal cord, which is caused by primary injury and subsequent progressive secondary injury (Colak et al., 2009; Dray et al., 2009). Secondary injury is a complex cascade reaction amplification process of self-destruction caused by primary injury, including ischemia and hypoxia, immune-inflammatory response, excitatory toxicity, free radical damage, lipid peroxidation, which was the main cause of aggravating neurological dysfunction and had reversibility and regulation (Ueno and Yamashita, 2008). Therefore, prevention and intervention of secondary injury is the core content of early SCI treatment (Boulenguez and Vinay, 2009).

In recent years, many scholars have confirmed that cell apoptosis, as well as cell necrosis, exist in the tissues after spinal cord injury. Cell necrosis is caused by primary injury, while cell apoptosis is caused by secondary injury (Yong et al., 1998). It was found that a large number of nerve cell apoptosis existed after SCI. And intracellular calcium ions ([Ca^2+^]i), plays an important role in the process of apoptosis. Previous studies have shown that [Ca^2+^]i is the initial messenger of Endoplasmic Reticulum Stress (ERS) reactive apoptosis, although the specific mechanism of ERS is largely unknown (Shen et al., 2002).

In 2000, Panchin found a coding sequence in vertebrates and named it Pannexin (Panx), which is homologous to the innexin gene of the invertebrate. There are three subtypes of Panx: Panx1, Panx2, and Panx3 (Panchin et al., 2000). Panx1 is abundantly expressed in many organs including the brain, spinal cord, bone (Penuela et al., 2007). Panx1 protein mainly functions by forming a homomer hemichannel (Locovei et al., 2006). And Panx1 channels are permeable to ions and molecules up to 1 kDa (Yang et al., 2020), which is large, non-selective plasma membrane channel that permit the movement of molecules and ions, including ATP to the extracellular space and Ca^2+^ release from ER (Vanden Abeele et al., 2006; Michalski et al., 2020). Vanden Abeele et al. demonstrated that Panx1 channels in the endoplasmic reticulum facilitates the movement of Ca^2+^ (Vanden Abeele et al., 2006). Yang et al. provided evidences that plasma membrane Panx1 channels were directly permeable to Ca^2+^, and facilitated increases in [Ca^2+^]i (Yang et al., 2020).

In the study of cerebral ischemia and anoxia injury, Thompson et al. also found that the Panx1 channel played an important role in cerebral ischemia and anoxia injury by regulating the transmission of intracellular and extracellular Ca^2+^ and ATP, and inducing neurocytes apoptosis and inflammatory response through various signal pathways (Thompson et al., 2006). However, a contrary evidence proposed by Harcha et al. showed that although panx1 channel located in the endoplasmic reticulum might be permeable to Ca^2+^, the cell membrane was not permeable to Ca^2+^ in the Hela cells. This led us to investigate the contribution of Panx1 to and its role in the Ca^2+^ overload and development of SCI *in vitro*. We reported here that Panx1 involved in the intracellular Ca^2+^ overload of SCI cells and promoted the apoptosis of spinal neurocytes.

## Materials and Methods

### Primary Spinal Neurocytes Culture

The embryonic spinal cord was dissected from embryonic day-15 (E15) Sprague-Dawley rat pups under the dissecting microscope (Olympus, Japan), and the spinal membrane and dorsal root ganglia were removed, followed by treatment with 0.15% trypsin (Gibco, United States) for 7 min at 37°C to detach the spinal neurocytes from primary tissues. Cells were washed three times with HBSS, suspended and centrifuged at 1,000rpm for 3min, and resuspended in NPC Medium and seeded on glass coverslips coated with poly-L-lysine in a 6-well plate at a density of 3 × 10^5^ cells/well. The cultures were maintained at 37°C under a humidified atmosphere of 5% CO_2_. This study was approved by the Institutional Animal Care and Use Committee of Fujian Medical University.

### Immunofluorescence Staining

We used immunofluorescence staining of neuronal nuclei (NeuN) and β-tubulin III to identify the primary spinal neurocytes and observed cell apoptosis by the staining of Hoechst33258. After culturing for 5–7 days, cells were fixed with 4% paraformaldehyde in PBS for 10min at room temperature and washed three times with PBS. Then the fixed cells were incubated with 1% BSA for 30 min to block the unspecific binding of the antibodies and permeabilized with 0.1% Triton X-100 solution in PBS for 10min at room temperature. Following by incubating cells in the primary antibody including anti-NeuN (1:200, ABclonal, United States) and anti-β-tubulin III (1:400, ABclonal, United States) overnight at 4°C. Alexa 594 goat anti-rabbit (1:1,000, Abcam, United Kingdom) and Alexa 488 goat anti-rat (1:1,000, Abcam, United Kingdom) was added and incubated at room temperature for 1 h to visualize NeuN and anti-β-tubulin III. Finally, the cell nuclei were stained with Hoechst33258 (Beyotime, China) for 15 min and observed under a fluorescence microscope.

### Establishment of the SCI Cell Model

Cells were seeded in a 96-well and treated with H_2_O_2_ (100, 300, 500, and 700 μM) in a serum-free NPC medium to establish the SCI cell model. The viability of spinal neurocytes was detected at 0.5, 1, 3, 5, and 7 h after H_2_O_2_ exposure using Cell Counting kit-8 (CCK-8) according to the manufacturer’s instruction. The optimal condition for establishing the SCI model was 700 μM H_2_O_2_ exposure for 3 h (data not shown).

### Lentivirus Mediated Overexpression and Knockdown of Panx1

Overexpression and knockdown of Panx1 were achieved using LV5-Panx1 and Sh-Panx1 recombinant lentivirus. The constructions of the recombinant lentivirus were as follows: GPLV5/EF-1αF/GFP+Puro (LV5) and GPLV3/H1/GFP+Puro (LV3) were purchased from Gene Pharma Co., Ltd. (Shanghai, China). The primer of Panx1 was designed and synthesized with the homologous sequences of NotI and BamHI added to the 5′ end of the forward and reverse primers by Sangon Co., Ltd. (Shanghai, China). The primer sequence was as follows: Primer (+): GGCCGCGCCACCATGGCCATCGCC, Primer (−): GATCCTTAGCAGGATGAATTCAGAAG. The Panx1 gene was amplified by PCR (ABI, United States), and the Panx1 gene fragment was recovered by gel electrophoresis. DNA endonuclease NotI and BamHI were used to perform enzyme digestion of LV5 at 37°C for 2 h. The linear carrier LV5 was recovered by a gel recovery kit. And then the ClonExpress^®^ Entry One Step Cloning Kit was used to clone Panx1 into a linearized LV5 vector. The reaction system was as follows: 5 × CE Entry Buffer 4 μl, LV5 1 μl, Panx1 2 μl, Exnase Entry 2 μl, ddH_2_O 11 μl. After being well mixed, the reaction was performed at 37°C for 30 min. The reaction tube was placed in an ice bath immediately after the reaction had been completed and cooled for 5 min. After that, the cloned products were transformed into the Top10 competent cells of E. coli, and LV5-Panx1 were extracted with the plasmid extract kit (TIANGEN, China), identified by enzyme digestion, and the positive clones were sent for sequencing. The Lenti Sh-Panx1 plasmid was produced similarly. The target sequence is GCTCCGACCTGAAGTTTAT- CA. GATCC was added to the 5′ end of the forward primer, which was complementary to the sticky end formed after BamHI enzyme digestion, and AATTC is added to the 5′ end of the reverse primer to compliment the sticky end formed by the EcoRI enzyme digestion. After the annealing reaction, a sh-DNA template with a concentration of 10 μM was obtained. Enzyme digestion of LV3 was performed by EcoRI and BamHI. Then the sh-DNA gene was cloned into the vector LV3. The reaction system was as follows: 10 × T4 Ligase Buffer 2 μl, LV3 1 μl, sh-DNA template (100 nM) 1 μl, T4 DNA Ligase (5 Weiss U/μl) 1 μl, ddH_2_O 15 μl. The reaction was performed at 22°C for 1 h. After transformation, the plasmids were extracted, identified by enzyme digestion, and sent to sequencing. LV5-Panx1 and Sh-Panx1 were co-transfected with the package plasmids (pGag/Pol, pRev, pvsv-g) into 293T cells to produce the recombinant lentivirus LV5-Panx1 and Sh-Panx1, respectively. Empty LV5 and LV3 plasmids were also co-transfected with the package plasmids into 293T cells to produce the LV-con and sh-con as control lentivirus. Virus titer and multiplicity of infections were detected. After the production of LV5-Panx1, Sh-Panx1 and control recombinant lentivirus, they were added to the medium of the primary spinal cord cells and the overexpression and knockdown of Panx1 in the cells were detected by western blot.

### Cell Viability Assay

The viability of spinal neurocytes was measured using the Cell Counting Kit-8 (Qiagen, United States) (QS) according to the manufacturer’s instructions. Briefly, cells were seeded on a 96-well plate at a density of 1 × 10^4^/ well, after the establishment of SCI and transfected with LV5-Panx1, Sh-Panx1, and control lentivirus, at 3, 8, 24, 48, and 72 h time points, the medium was removed, and 10 μl CCK-8 reagent in 90 μl medium were added. Following incubated at 37°C for 2 h, the absorbances were read at 450 nm on the microplate reader (Bio-Rad, United States). All experiments were run in duplicate and were repeated at least three times.

### Flow Cytometry

Cells (NC, LV-con, sh-con, LV5-Panx1, and sh-Panx1) were seeded on a 6-well plate at a density of 1 × 10^6^/ well. At 24 h after H_2_O_2_ exposure, cell apoptosis was detected by an annexin V-apoptosis kit. The cells were washed and incubated for 15 min at room temperature in the presence of annexin V-APC and propidium iodide (PI). A flow cytometer (Accuri C6, United States) was used for detection. PI fluorescence was performed using the FL2 channel, while AnnexinV-APC fluorescence was performed using the FL4 channel. 20,000 cells in the portal were collected and apoptosis was analyzed in each group.

### Intracellular Ca^2+^ Detection

Primary neurocytes were seeded in a 28.2 mm dish. At 24 h after H_2_O_2_ exposure, the BBcellProbeTMF04 probe (1:200) was added and incubated at 37°C for 40 min. Laser confocal microscopy (Carl Zeiss, Germany) was used to detect intracellular Ca^2+^ signal in each group. The change of Ca^2+^ signal was inversely proportional to the intensity of red fluorescence. Image J was used for semi-quantitative analysis of fluorescence intensity in each group.

### Real-Time PCR (RTPCR)

The cells were diluted to 1 × 10^5^ cell/mL and were incubated for 24 h in six-well plates (Falcon, BD Biosciences). After the establishment of SCI and transfected with LV5-Panx1, Sh-Panx1, and control lentivirus for 24 h. Total RNA of rat spinal neurocytes was extracted by Trizol (Invitrogen, United States). The PrimeScript^TM^ RT reagent Kit with gDNA Eraser Kit (TaKaRa, Japan) was used to conduct a Reverse Transcription reaction to synthesize cDNA. The primers of β-actin, Bcl-2, Bax, Caspase3, PARP1 were designed and synthesized by Sangon Co., Ltd. (Shanghai, China), and the sequence was shown in the Table 1. Real-time PCR reaction solution was prepared according to the instruction of SYBR^®^ Premix Ex Taq^TM^ (Tli RNaseH Plus) (TaKaRa, Japan). Reaction conditions were set on the fluorescence quantitative PCR apparatus (LightCycler/LightCycler480 System, Roche, Switzerland): denaturation at 95°C for 30 s. PCR at 95°C for 5 s, 60°C for 30 s, 40 cycles. Dissolution at 95°C for 5 s, 60°C for 1 min. The Ct value of the target gene and β-actin of each group was generated by the fluorescence quantitative PCR apparatus, and each gene expression of relative quantity was calculated by the formula: *F* = 2^–ΔΔCt^, and the normal cells were used as the control.

**TABLE 1:** Primer sequence of β-actin, Bax, Bcl-2, Caspase3, PARP1.

Gene	Primer	bp
β-actin	F: ACAGGATGCAGAAGGAGATTAC	117
	R: ACAGTGAGGCCAGGATAGA	
Bax	F: GATGGCCTCCTTTCCTACTTC	96
	R: CTTCTTCCAGATGGTGAGTGAG	
Bcl-2	F: GTGGATGACTGAGTACCTGAAC	125
	R: GAGACAGCCAGGAGAAATCAA	
Caspase3	F: ACAGTGGAACTGACGATGATATG	109
	R: TCCCTTGAATTTCTCCAGGAATAG	
PARP1	F:CTTGGTGGAGTACGAGATTGAC	160
	R:GGTGTAGAAGCGATTGGAGAG	

### Western Blot

The levels of Bcl-2, Bax, Caspase3, and PARP1, regulatory genes involved in the intracellular Ca^2+^ homeostasis, and apoptosis pathways, were determined by western blot analysis. Cells were harvested and lysed for western blot analysis. Lysates were prepared as previously described (Zhang et al., 2019). Membranes were incubated with the following primary antibodies: anti Bcl-2 (1:800, ABclonal, United States), anti Bax (1:800, ABclonal, United States), anti Caspase3 (1:1,000, ABclonal, United States), anti PARP1 (1:1,000, ABclonal, United States). HRP-conjugated secondary Abs were applied, and a supersensitive ECL Chemiluminescence Kit (Beyotime, China) was used to detect proteins. anti-β-actin (1:1,600, ABclonal, United States) was used as a loading control.

### Statistical Analysis

Data are analyzed using SPSS 20.0 software (SPSS Inc., Chicago, IL, United States). All experiments were performed at least three times. Data were presented as the mean ± SD. Student’s *t*-test and/or ANOVA were performed for statistical analyses, and probability values < 0.05 indicated statistically significant differences.

## Results

### SCI of the Rat Primary Spinal Neurocytes After Oxidative Stress Was Increased by Panx1 Overexpression and Attenuated by Panx1 Depletion, *in vitro*

**FIGURE 1:**
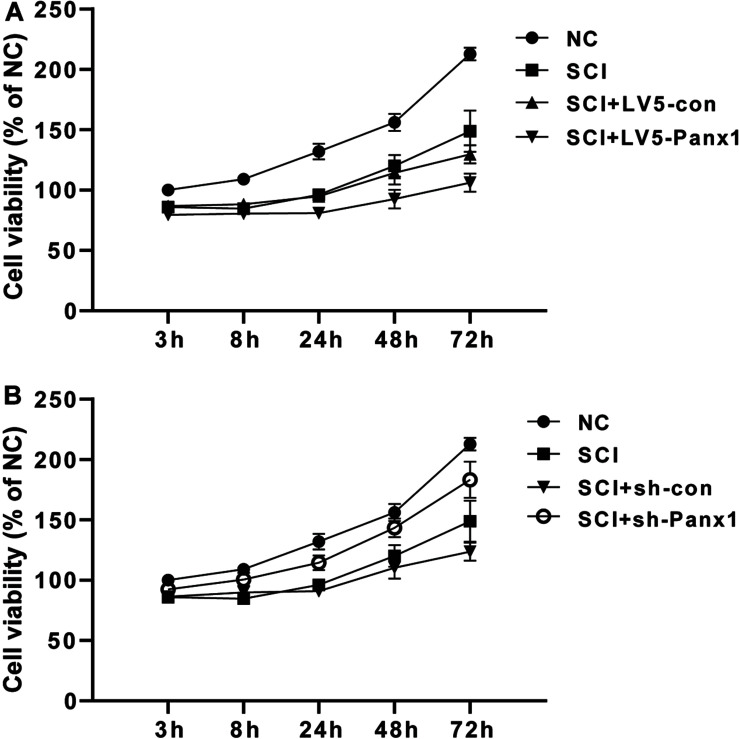
Cell viability of the rat primary spinal neurocytes SCI model was reduced by Panx1 overexpression and increased by Panx1 depletion, *in vitro*. **(A)** SCI primary spinal neurocytes showed reduced cell viability when compared with normal cells at 24 h, 48 h, and 72 h time points after H_2_O_2_ treatment. Panx1 overexpression (LV5-Panx1) further reduced cell viability when compared with control cells (LV-con) and normal cells after H_2_O_2_ treatment. **(B)** Panx1 depletion (sh-Panx1) promoted cell viability at 24, 48, and 72 h time points after H_2_O_2_ treatment when compared with control cells (sh-con) and normal cells after H_2_O_2_ treatment.

SCI primary spinal neurocytes showed reduced cell viability when compared with normal cells at 24, 48, and 72 h time points after H_2_O_2_ treatment. Panx1 overexpression (LV5-Panx1) further reduced cell viability when compared with control SCI cells (SCI + LV-con) and SCI cells (Figure 1A). On the other hand, Panx1 depletion reversed the cell injury and the Panx1 depletion cells showed increased cell viability when compared with control SCI cells (SCI + sh-con) and SCI cells (Figure 1B).

### Panx1 Decreased Cell Viability, Induced Upregulation of Apoptosis in the SCI Cells, *in vitro*

**FIGURE 2:**
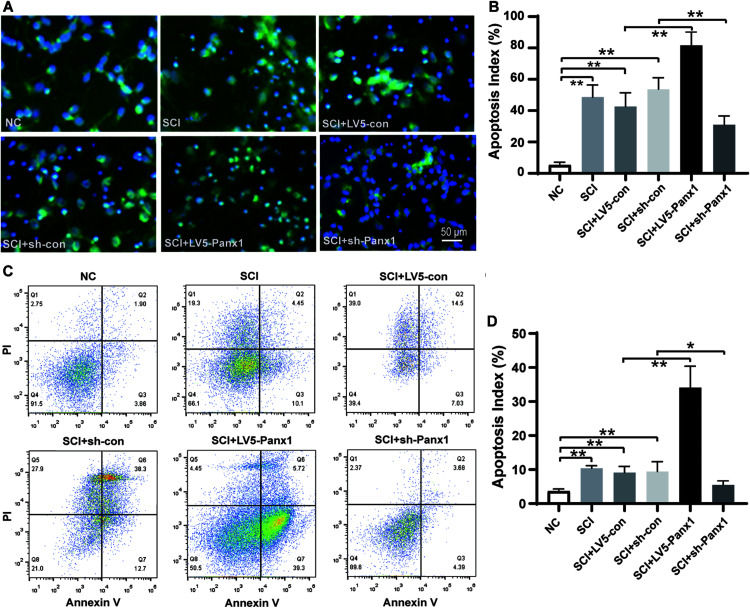
Panx1 increased cell apoptosis in the rat primary spinal neurocytes SCI model, *in vitro*. **(A)** Cell apoptosis induced by H_2_O_2_ was measured by Hoechst33258 staining under fluorescence microscopy, β-tubulin III served as a neuron cell surface maker. The nuclei of apoptotic cells were dense or fragmented. Increased number of apoptotic cells were shown in SCI model comparing with untreated normal cells, Panx1 overexpression (LV5-Panx1) further increased the number of apoptotic cells at 24 h time point after H_2_O_2_ treatment, while Panx1 depletion reverses the effect when compared with control cells (LV-con, sh-con) and normal cells after H_2_O_2_ treatment. **(B)** The apoptotic index was measured as the percentage of apoptotic cells and bodies per all cells. mean ± SD was used, n = 5, *p < 0.05, **p < 0.01. **(C)** Annexin V/Propidium Iodide (PI) apoptosis analysis demonstrated an increased apoptotic cell population in the SCI cells at 24 h time point after H_2_O_2_ treatment, Panx1 overexpression (LV5-Panx1) further increased the number of apoptotic cells at 24 h time point after H_2_O_2_ treatment, while Panx1 depletion reverses the effect when compared with control cells (LV-con, sh-con) and normal cells after H_2_O_2_ treatment. **(D)** The apoptotic index was measured using flow cytometry (Q3), mean ± SD was used, *n* = 3, **p < 0.01.

Cell apoptosis induced by H_2_O_2_ was measured by Hoechst33258 staining under fluorescence microscopy (Figures 2A,B) and flow cytometry (FCM) with Annexin V-APC/PI staining (Figures 2C,D). Increased number of apoptotic cells were shown in SCI model comparing with untreated normal cells (Figure 2A), Panx1 overexpression (LV5-Panx1) further increased the number of apoptotic cells at 24 h time point after H_2_O_2_ treatment, when compared with control cells (LV-con) and normal cells after H_2_O_2_ treatment. On the other hand, a reduced number of apoptotic cells in SCI model cells with Panx1 depletion (sh-Panx1) were observed when compared with cells transfected with sh-con or SCI model cells (Figures 2A,B). Our finding suggested that Panx1 increased the cell apoptosis of the primary spinal neurocytes after H_2_O_2_ treatment, therefore increased SCI in the cells, *in vitro.* Besides, Annexin V apoptosis analysis demonstrated an increased apoptotic cell population in the Panx1 overexpression cells compared with cells transfected with LV-con or treated with a vehicle. On the other hand, a reduced number of apoptotic cells in the SCI model with Panx1 depletion (sh-Panx1) were observed when compared with cells transfected with sh-con or treated with vehicle, this suggested Panx1 increased cell apoptosis in the rat primary spinal neurocytes SCI model, *in vitro* (Figures 2C,D). Together, we showed that Panx1 decreased cell viability, induced upregulation of apoptosis in the SCI model, *in vitro.*

### Panx1 Increased Intracellular Ca^2+^ of SCI Cells

**FIGURE 3:**
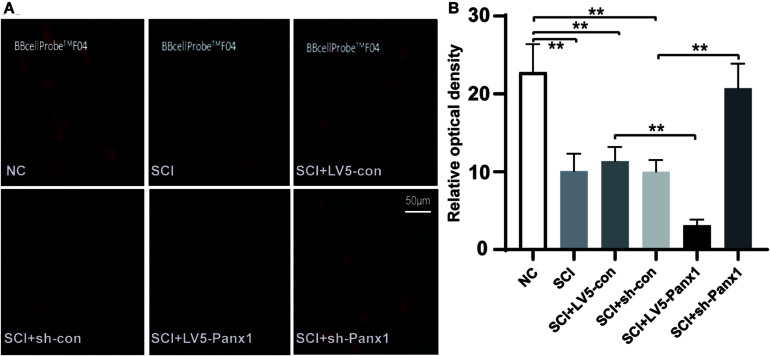
Panx1 increased the intracellular Ca^2+^ signal of SCI cells. **(A)** We detect the intracellular Ca^2+^ signal with a confocal microscope. The red fluorescence intensity was inversely proportional to that of the intracellular Ca^2+^ signal. Normal cells without treatment served as control. Elevated intracellular Ca^2+^ signal was shown in SCI model cells comparing with untreated normal cells. Panx1 overexpression (LV5-Panx1) enhanced the intracellular Ca^2+^ level while Panx1 depletion reversed this effect compared with control cells (LV-con, sh-con) and normal cells at 24 h time point after H_2_O_2_ treatment. **(B)** A densitometric quantification of optical density was performed by Image J and expressed as IntDen relative to the area. It was inversely proportional to that of the intracellular Ca^2+^ level. Mean ± SD was used, *n* = 3, **p < 0.01.

Increased intracellular Ca^2+^ is linked to neuronal apoptosis. We detect intracellular Ca^2+^ signal with a confocal microscope. The red fluorescence intensity was inversely proportional to the change of the intracellular Ca^2+^ signal. Our results showed elevated intracellular Ca^2+^ signal in SCI model cells comparing with untreated normal cells. Panx1 overexpression (LV5-Panx1) enhanced the intracellular Ca^2+^ signal while Panx1 depletion reversed this effect compared with control cells (LV-con, sh-con) and normal cells at 24 h time point after H_2_O_2_ treatment (Figure 3). This finding suggested that Panx1 increased cell apoptosis via the upregulation of intracellular Ca^2+^ in the SCI model, *in vitro.*

### Panx1 Increased the Levels of Bax, Caspase-3 and PARP1 but Suppressed the Level of Bcl-2

**FIGURE 4:**
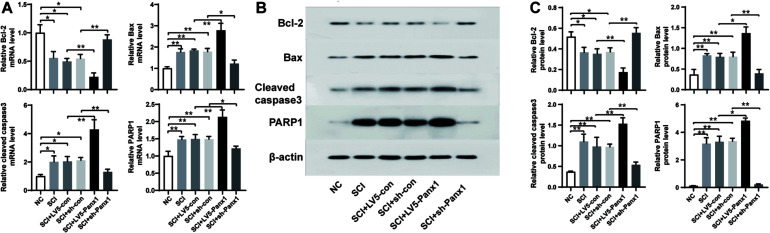
Panx1 increased the level of Bax, cleaved Caspase-3 and PARP1 but suppressed the level of Bcl-2. mRNA and protein level of Bcl-2, Bax, Caspase-3, and PARP1were analyzed by RT-PCR **(A)** and western blot **(B)** at 24 h time point after H_2_O_2_ treatment. **(C)** Quantification of protein bands using ImageJ Gel Analysis program. SCI cells showed elevated mRNA and protein level of proapoptotic protein Bax, and apoptosis pathway proteins including cleaved Caspase-3 and PARP1, as well as the decreased level of antiapoptotic protein Bcl-2. Panx1 overexpression (LV5-Panx1) further enhanced the level of Bax, Caspase-3 and PARP1, but suppressed the level of Bcl-2, when compared to SCI cells or SCI cells transfected with LV-con. On the other hand, Panx1 depletion (sh-Panx1) reduced the levels of Bax, Caspase-3 and PARP1 but increased the level of Bcl-2, when compared to SCI cells or SCI cells transfected with sh-con. Mean ± SD was used, *n* = 3, * *p* < 0.05, **p < 0.01.

SCI cells showed elevated mRNA and protein levels of proapoptotic protein Bax, and apoptosis pathway proteins including cleaved Caspase-3 and PARP1, as well as the decreased levels ofantiapoptotic protein Bcl-2. Panx1 overexpression (LV5-Panx1) further enhanced the level of Bax, Caspase-3 and PARP1, but suppressed the level of Bcl-2, when compared to SCI cells or SCI cells transfected with LV-con. On the other hand, Panx1 depletion (sh-Panx1) reduced the levels of Bax, Caspase-3 and PARP1 but increased the level of Bcl-2, when compared to SCI cells or SCI cells transfected with sh-con (Figure 4).

### Identification of Isolated Spinal Primary Neurocytes

To identify the spinal primary neurocytes, we used immunofluorescence staining of specific NeuN and tubulin-III to label the cells. NeuN and tubulin-III double-positive cells were identified as the spinal primary neurocytes. Hoechst33258 served as the nuclear counterstain. The percentage of spinal cord neurocytes in the isolated primary cells was identified to be 90% (Supplementary Figure 1).

## Discussion

In this study, we established the SCI model of rat primary spinal cord cells. We showed that decreased cell viability and apoptosis were seen in SCI cells, which was further enhanced under Panx1 overexpression and mitigated by Panx1 deficiency. H_2_O_2_ induced an increase of intracellular Ca^2+^ signal and upregulated levels of Bax, Caspase-3, and PARP1, which was enhanced by Panx1 overexpression or attenuated by Panx1 depletion. The results indicated that Panx1 involved in the intracellular Ca^2+^ overload of SCI cells, which led to endoplasmic reticulum stress (ERS) and promoted the apoptosis of spinal neurocytes through the endoplasmic reticulum pathway, thus aggravating the secondary injury of SCI.

Nerve cell apoptosis was frequently seen after SCI. Glial cell apoptosis was observed in the damaged area at 4–9 days after SCI (Li et al., 1996, 2000). And Crowe et al. (1997) [1][5] reported a large number of apoptotic cells in the degenerative nerve fiber bundles of monkeys. Apoptosis of neurocytes was found around the damaged area in the spinal cord of patients who died of SCI in 3 h to 2 months, with degenerative changes occurred in adjacent segments (Emery et al., 1998). Besides, studies have confirmed that neurocytes necrosis was dominant in the early stage of SCI in rats, and the apoptosis of neurocytes began in 6 h after injury, which could last for 2 weeks or even longer (Liu et al., 2020). Panx1 was correlated with the inflammatory response (Pelegrin and Surprenant, 2006; Orellana et al., 2013), Pyroptosis (Kayagaki et al., 2011, 2013; Hagar et al., 2013; de Gassart and Martinon, 2015; Yang et al., 2015), and autophagy (Ayna et al., 2012; Martins et al., 2014) after nervous system injury, which promoted apoptosis in the cells. These studies in line with our data that showed overexpression of Panx1 increased the apoptosis rate in SCI cells, which were migrated by Panx1 depletion. Therefore, it was revealed that Panx1 was connected to the apoptosis of nerve cells after SCI.

Ca^2+^ was linked to neuronal cell apoptosis. The increased intracellular Ca^2+^ in neurons could trigger the arachidonic acid metabolism cascade and activate the xanthine oxidation system through oxidative stress (Lukic-Panin et al., 2007), causing the formation of a large number of free radicals, forming a vicious cycle, and finally activating the Ca^2+^ dependent endonuclease, initiating the neuronal apoptosis process, leading to neuronal apoptosis (Guven et al., 2011). The activated Panx1 channel could result in increased intracellular Ca^2+^. Yang et al. (2020) provided several lines of evidence that suggested that plasma membrane Panx1 channels were directly permeable to Ca^2+^, and facilitated increases in [Ca^2+^]i. These included no Ca^2+^ response to TNF when Panx1 was silenced by siRNA or the channel was inhibited using the Panx1 specific inhibitor peptide PxIL2P. In addition, increases in [Ca^2+^]i are associated with IL-1β -synthesis, that can be ablated following reductions in [Ca^2+^]i by blocking the Panx1 channel (Yang et al., 2020). These evidences support our data that showed Panx1 overexpression promoted [Ca^2+^]i and resulted in cell apoptosis. While depletion of Panx1 improved the viability of neurocytes after SCI and reduced [Ca^2+^]i, therefore had a protective effect on oxidative stress injury of neurocytes.

Bcl-2 and Bax were two typical regulatory genes (Kale et al., 2018; Kalkavan and Green, 2018), which participated in the maintenance of intracellular Ca^2+^ homeostasis. Bcl-2 exerted a direct effect on the Ca^2+^ handling in the ER by regulating the movement of Ca^2+^ through the ER membrane. Overexpression of proapoptotic Bax or knock-down Bcl-2 increased Ca^2+^ and augmented the number of releasable ER Ca^2+^ (Xu et al., 2008; Chiu et al., 2018). However, Bax is mostly distributed in the cytoplasm in the form of an inactive monomer through forming a heterodimer with Bcl-2, which blocked the anti-apoptotic function of Bcl-2 (Hetz et al., 2006). Therefore, the ratio of Bcl-2 to Bax is of great significance in determining whether the cells enter the state of apoptosis.

Caspase-3 was the main effector Caspase, a common downstream effector protein of various apoptosis pathways, which played a central role in the apoptosis process and was known as the “death executive protease” (Rogers et al., 2017). When the Ca^2+^ homeostasis of the endoplasmic reticulum was disrupted or excessive protein accumulation in the endoplasmic reticulum, Caspase-12 might indirectly activate caspase-3, which was triggered by ERS, but the specific mechanism is not clear and needs to be confirmed by further studies (Lamb et al., 2006; Tan et al., 2006; Kim et al., 2008). Activated cleaved-Caspase-3 cleaved the DNA repair protein PARP-1 in the nucleus, causing cell disintegration and eventually leading to cell apoptosis (Alano et al., 2010; Ba and Garg, 2011). Our results demonstrated SCI activated Panx1, which led to the increase of intracellular Ca^2+^. B-cell lymphoma-2 (Bcl-2) family of proteins, including pro-apoptotic and anti-apoptotic members as well, associated with the neuronal Ca^2+^ homeostasis and mediated the intrinsic apoptosis pathway by controlling mitochondrial outer membrane (MOM) integrity. Bax as one of the “pro-apoptotic” Bcl-2 family proteins played a critical role in the apoptosis pathway after the neuronal injury (D’Orsi et al., 2017). Our results indicated that increased intracellular Ca^2+^ after SCI resulted in elevated Bax level in spinal cord cells, which bonded to the antiapoptotic protein Bcl-2, decreased its level, and initiated the intrinsic apoptosis pathway, including the increase of cleaved caspase-3, and PARP-1.

## Conclusion

Our study demonstrated for the first time that Panx1 increased cell apoptosis in SCI spinal cord cells *in vitro.* This effect was associated with intracellular Ca^2+^ overload, and in turn, activated the cell apoptosis pathways. Our findings suggested that the Panx1 channel was a promising new therapeutic target for the treatment of SCI. One of the limitations in the present study is that we cannot identify whether the increase intracellular Ca^2+^ induced by SCI is from extracellular Ca^2+^ or Ca^2+^ release from ER. Further studies are needed to identify the mechanisms of Panx1 channel mediated control of [Ca^2+^]i.

## Data Availability Statement

The original contributions presented in the study are included in the article/Supplementary Material, further inquiries can be directed to the corresponding author/s.

## Author Contributions

YH designed and performed the experiments, interpreted the results, and prepared the manuscript. JinL and XC helped in performing experiments. JiaL conceived, designed, and oversaw the project. All authors have read and approved the manuscript for publication.

## Conflict of Interest

The authors declare that the research was conducted in the absence of any commercial or financial relationships that could be construed as a potential conflict of interest.

## Supplementary Material

Supplementary Figure 1 Identification of isolated spinal primary neurocytes. The spinal primary neurocytes were isolated from rat embryos. **(a,b)** NeuN and tubulin-III staining double-positive cells were identified as the spinal primary neurocytes. **(c)** Hoechst33258 served as the nuclear counterstain. **(d)** Merged image of **(a–c)**. The percentage of spinal cord neurocytes in the isolated primary cells was 90%.
