# Supplementary figures and images for: Pannexin-1 Contributes to the Apoptosis of Spinal Neurocytes in Spinal Cord Injury
Source: Front Physiol. 2021 Apr 27;12:656647. doi: 10.3389/fphys.2021.656647 (PMC8112589; doi:10.3389/fphys.2021.656647)

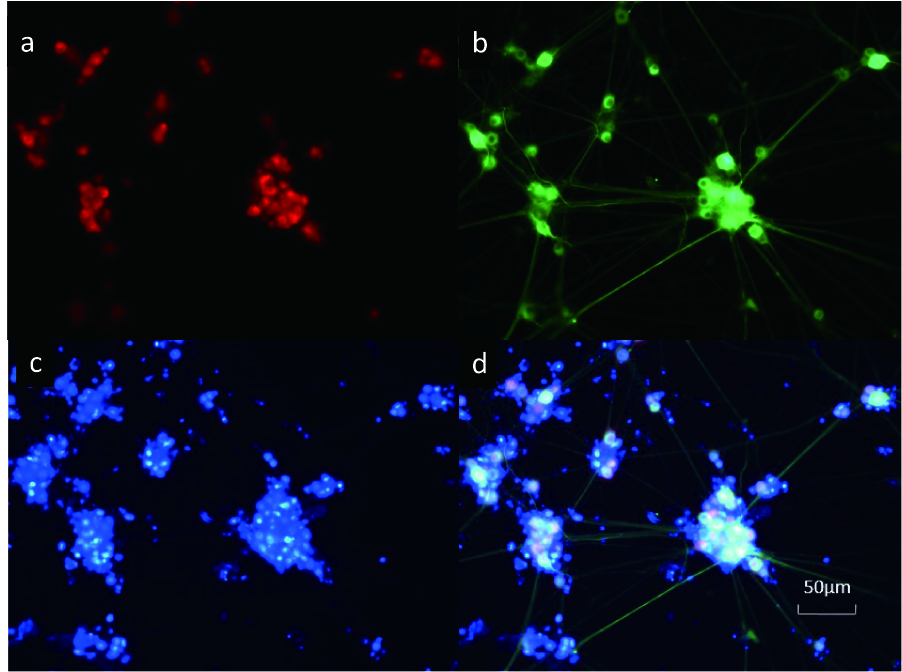

Supplement: Supplementary Figure 1 — Identification of isolated spinal primary neurocytes. The spinal primary neurocytes were isolated from rat embryos. (a,b) NeuN and tubulin-III staining double-positive cells were identified as the spinal primary neurocytes. (c) Hoechst33258 served as the nuclear counterstain. (d) Merged image of (a–c). The percentage of spinal cord neurocytes in the isolated primary cells was 90%. [file Image_1.TIF]
